# Applications of Single-Cell Omics to Dissect Tumor Microenvironment

**DOI:** 10.3389/fgene.2020.548719

**Published:** 2020-11-27

**Authors:** Tingting Guo, Weimin Li, Xuyu Cai

**Affiliations:** ^1^Institute of Respiratory Health, Frontiers Science Center for Disease-related Molecular Network, West China Hospital, Sichuan University, Chengdu, China; ^2^Precision Medicine Research Center, West China Hospital, Sichuan University, Chengdu, China; ^3^Department of Respiratory and Critical Care Medicine, West China Hospital, Sichuan University, Chengdu, China; ^4^The Research Units of West China, Chinese Academy of Medical Sciences, West China Hospital, Chengdu, China; ^5^Precision Medicine Key Laboratory of Sichuan Province, Chengdu, China

**Keywords:** single-cell sequencing, single-cell multi-omics, tumor microenvironment, immunotherapy, tumor-specific immunity, tumor infiltrating lymphocytes (TILs), tumor infiltrating myeloid cells (TIMs)

## Abstract

The recent technical and computational advances in single-cell sequencing technologies have significantly broaden our toolkit to study tumor microenvironment (TME) directly from human specimens. The TME is the complex and dynamic ecosystem composed of multiple cell types, including tumor cells, immune cells, stromal cells, endothelial cells, and other non-cellular components such as the extracellular matrix and secreted signaling molecules. The great success on immune checkpoint blockade therapy has highlighted the importance of TME on anti-tumor immunity and has made it a prime target for further immunotherapy strategies. Applications of single-cell transcriptomics on studying TME has yielded unprecedented resolution of the cellular and molecular complexity of the TME, accelerating our understanding of the heterogeneity, plasticity, and complex cross-interaction between different cell types within the TME. In this review, we discuss the recent advances by single-cell sequencing on understanding the diversity of TME and its functional impact on tumor progression and immunotherapy response driven by single-cell sequencing. We primarily focus on the major immune cell types infiltrated in the human TME, including T cells, dendritic cells, and macrophages. We further discuss the limitations of the existing methodologies and the prospects on future studies utilizing single-cell multi-omics technologies. Since immune cells undergo continuous activation and differentiation within the TME in response to various environmental cues, we highlight the importance of integrating multimodal datasets to enable retrospective lineage tracing and epigenetic profiling of the tumor infiltrating immune cells. These novel technologies enable better characterization of the developmental lineages and differentiation states that are critical for the understanding of the underlying mechanisms driving the functional diversity of immune cells within the TME. We envision that with the continued accumulation of single-cell omics datasets, single-cell sequencing will become an indispensable aspect of the immune-oncology experimental toolkit. It will continue to drive the scientific innovations in precision immunotherapy and will be ultimately adopted by routine clinical practice in the foreseeable future.

## Introduction

A tumor grows within a highly complex and dynamic local environment composed of immune cells, stromal cells, endothelial cells, as well as other non-cellular components such as secreted cytokines, chemokines and extracellular matrix (ECM). This complex ecosystem is collectively termed as the tumor microenvironment (TME), characterized by its profound heterogeneity, plasticity, and complex cross-interaction (Fridman et al., 2017). Our growing understanding of the TME has laid scientific foundation for cancer immunotherapy, which is arguably one of the greatest breakthroughs in cancer therapeutics over the past decade (Hodi et al., 2010; Robert et al., 2014; Garon et al., 2015; Ribas and Wolchok, 2018). The clinical success on immune checkpoint blockade (ICB) therapies has shifted the cancer therapeutic paradigm by demonstrating the great importance of T-cell mediated anti-tumor immunity; therefore, the TME has become a prime target for future anti-tumor therapies (Sharma and Allison, 2015; Wei et al., 2018). The recent technical and computational advances on single-cell sequencing provide a powerful tool to profile the TME cellular landscape in an unbiased and comprehensive way, which is enabling researchers to further dissect the cellular and molecular mechanisms underlying tumor-specific immune responses, tumor-immune cell interactions and immune evasion. Recent studies applying single-cell RNA sequencing (scRNA-seq) on the TME have yielded unprecedented resolution of its cellular and molecular complexity, which is likely to form a key determining factor in tumor progression and therapeutic response (Giladi and Amit, 2018; Papalexi and Satija, 2018; Ren et al., 2018; Gomes et al., 2019). We envision that single-cell sequencing will become an indispensable aspect of the immune-oncology experimental toolkit. With the continued accumulation of single-cell transcriptomics datasets and further extension to single-cell genomics, epigenomics and proteomics, we are geared to resolve not only the cellular composition and functional states of immune cells, but also the developmental history, regulatory network and cellular interactions of tumor and immune cells. Such increasing knowledge of the TME will pave the way for future precision immunotherapy through the establishment of connection between the TME within each patient’s tumor and the corresponding response to immunotherapy, and thus facilitate the design of optimal therapeutic strategies tailored to each patient. Meanwhile, unknown immune cell subtypes and states in the TME could be identified with the help of single-cell sequencing to yield novel targets for the development of future immunotherapy. In the review, we will discuss the recent advances on understanding the diversity of TME and its functional impact on tumor progression and immunotherapy response driven by single-cell sequencing, with the primary focus on the major immune cell types identified within the TME.

## Recent TME Studies Driven by Single-Cell RNA Sequencing

### Tumor Infiltrating Lymphocytes

Single-cell RNA sequencing has been widely used to profile the intratumoral immune landscape of various types of tumors, including skin, breast, lung, liver, colorectal, head and neck, brain, bladder, renal, and endometrial cancers (Table 1; Tirosh et al., 2016; Lavin et al., 2017; Müller et al., 2017; Puram et al., 2017; Zheng et al., 2017; Azizi et al., 2018; Guo et al., 2018; Jerby-Arnon et al., 2018; Lambrechts et al., 2018; Sade-Feldman et al., 2018; Savas et al., 2018; Zhang et al., 2018, 2019, 2020; Clarke et al., 2019; Li H. et al., 2019; Yost et al., 2019; Zilionis et al., 2019; Cillo et al., 2020; Oh D.Y. et al., 2020; Wu et al., 2020). Tumor infiltrating lymphocytes (TILs) have been the primary focus in many early studies since they are considered as the major immune cell type responsible for tumor-specific immunity. Accordingly, the level of T cell infiltration has been most frequently correlated with good prognosis in multiple cancer types (Fridman et al., 2017; Binnewies et al., 2018; Galon and Bruni, 2020). Immune checkpoint blockade therapy functions to restore the T cell-mediated anti-tumor immune responses by blocking the checkpoint receptors, such as PD-1 and CTLA-4, which are expressed in T cells, or their ligands, such as PD-L1, which is upregulated in tumor and antigen presenting cells (APCs) upon chronic antigen stimulation within the TME (Chen and Mellman, 2017; Wei et al., 2018; Callahan and Wolchok, 2019). Although the ICB therapy has achieved remarkable and durable clinical responses on selected patients and cancer types, its overall response rate has been limited, and many patients with initial response suffer from refractory disease or acquired resistance. The observed variation in ICB efficacy has been linked to various aspects of the TME, particularly correlated with signatures of the intratumoral T cell states, including overall T cell infiltration, activation and exhaustion (Sharma et al., 2017; Binnewies et al., 2018).

**TABLE 1:** Recent single-cell sequencing studies on human tumor microenvironment.

Cancer type	Literature	Patient characteristics	Sample type	Cell type	Cell number	Data type	Platform	Major conclusion
Melanoma	Tirosh et al. (2016)	Nineteen patients with diverse treatment background	L, T	CD45+ and CD45-	4645 cells	scRNA-seq, bulk WES	SMART-seq2	Identified activation-dependendent T cell exhaustion and exhaustion-associated T cell expansion
Melanoma	Jerby-Arnon et al. (2018)	Thirty-one pre- and post-ICB treated patients	T	All TME cell types	7189 cells	scRNA-seq, bulk RNA-seq	SMART-seq2, 10X Genomics	Identified overlapping ICB resistance program and post-ICB treatment program expressed by malignant cells: associated with T cell exclusion, down-regulation of APC and IFN-g pathway
Melanoma	Sade-Feldman et al. (2018)	Thirty-two pre- and post-ICB treated patients	T, longitudinal biopsies	CD45 + immume cells	16291 cells	scRNA-seq, bulk WES, bulk TCR repertoire	SMART- seq2	Identified two unique states of CD8 + TILs: TCF7 + memory-like T cells predicted positive clinical outcome, and CD39 + exhausted-like T cells predicted negative clinical outcome
Melanoma	Li H. et al. (2019)	Thirty-four patients with diverse treatment background	T	All TME cell types	46612 cells	scRNA-seq, scTCR repertoire	MARS-seq	Identified two separate lineage of CD8 + TILs: bystander cytotoxic T cells and dysfunctional T cells; dysfunctional CD8 + T cells are the major intratumoral proliferative cells and the intensity of the dysfunctional signature is associated with tumor reactivity
BCC & SCC	Yost et al. (2019)	Eleven BCC and 4 S CC pre- and post-ICB treated patients	T	All TME cell types	79046 cells	scRNA-seq, scTCR repertoire, bulk RNA-seq	10X Genomics	CD39 + tumor-reactive TILs are dysfunctional and clonally expanded; increased T cell infiltration and decreased mutational load following ICB treatment, whereas pre-existing tumor-reactive TILs have limited reinvigoration capacity and are replaced by novel clones from PBMC after treatment
NSCLC	Lavin et al. (2017)	Eighteen treatment naïve patients	B, N, T	CD45+ CD3- non- lymphocyte immune cells	1473 cells	scRNA-seq, CyToF, bulk RNA-seq and TCR	MARS-seq	Identified enrichment of T, B lymphocytes, TAM, and depletion of NK and CD141 + DC in TME compared to normal tissue; CD8 + PD-1 + T cells are clonally expanded and T effector to Treg ratio are reduced in TME
NSCLC	Guo et al. (2018)	Fourteen treatment naïve patients	B, N, T	CD8, CD4CD25(hi), CD4CD25(lo) T cells	12346 cells	repertoir scRNA-seq, scTCR repertoire	SMART-seq2	Identified inter-tissue effector T cells with migratory nature; identified two distinct Treg populations; patients enriched for “pre-exhaused” CD8+ T cells and TNFRSF9- non-activated Tregs associated with better prognosis
NSCLC	Lambrechts et al. (2018)	Eight treatment naïve patients	N, T	All TME cell types	52698 cells discovery, 40250 cells validation	scRNA-seq	10X Genomics	Identified fibroblast expressing different collagen sets, endothelial cells downregulating immune cell homing and genes co-regulated with established immune checkpoint transcripts and correlating with T cell activity
NSCLC	Clarke et al. (2019)	Twelve treatment naïve, 2 pre- and N, T post-ICB treated patients	CD103 + and CD103- CD8 T cells	N, T	∼12000 cells	scRNA-seq, scTCR repertoire, bulk RNA-seq, TCR repertoire, ATAC-seq	SMART-seq2, 10X Genomics	Identified a CD103 + PD-1 + TIM3 + IL7R- Trm s ubset enriched in tumor; this subset is tumor-reactive, proliferative and cytotoxic, and expands in response to ICB treatment
NSCLC	Zilionis et al. (2019)	seven treatment naïve patients	B, N, T	All TME cell types	54773 cells	scRNA-seq	inDrop	Mapped tumor-infiltrating myeloid cells landscape, identified profound diversity within each cell lineage but large concordance between human and mouse
Breast	Azizi et al. (2018)	Eight treatment naïve patients (ER, PR, Her2, TNBC)	B, L N, T	CD45 + immume cells	47016 cells from inDrop, 27000 from 10XG	scRNA-seq, scTCR repertoire	i nDrop, 10X Genomics	Identified expanded diversity of T cell population in tumor compared to normal tissue, defined by continous spectrum of activation and terminal differentation level; TCR clonotypes and cell state diversity both contribute to intratumoral heterogeneity of T cell populations
Breast	Savas et al. (2018)	Two treatment naïve TNBC patients	T	T cells	6311 cells	scRNA-seq	10X Genomics	Identified CD103 + tissue-resisdent memory T cells co-expressing immune checkpoint molecules and effector proteins, and assoicates with improved patient survival
CRC	Zhang et al. (2018)	Twelve treatment naïve patients (8 B, N, T MSS, 4 MSI)	B, N, T	CD8, CD4CD25(hi), CD4CD25(lo) T cells	11138 cells	scRNA-seq, scTCR repertoire	SMART-seq2	Identified MSI-enriched Th-1-like subset; identified CD8 + effector and exhausted T cells subsets both with high level of clonal expansion but developed from separate lineages
CRC	Zhang et al. (2020)	Eighteen treatment naïve patients	B, N, T	CD45+ and CD45-	43817 cells from 10XG, 10468 cells from SMART-seq2	scRNA-seq, scTCR repertoire	SMART-seq2, 10X Genomics	Identified two distinct subsets of tumor associated macrophages (TAM), which showed differential sensivitity to CSF1R blockade; identified cell type specfic responses following CD40 blockade, including cDC1 specific activation, Th1 and CD8 + memory T cell specific expansion
HCC	Zheng et al. (2017)	Six treatment naïve patients	B, N, T	CD8, CD4CD25(hi), CD4CD25(lo) T cells	5063 cells	scRNA-seq, scTCR repertoire	SMART-seq2	Exhausted CD8 + T cells and Tregs are enriched and cloncally expanded in HCC; LAYN is upregulated on activated CD8 + T cells and Tregs and surppresses the CD8 + T cell functions
HCC	Zhang et al. (2019)	Sixteen treatment naïve patients	B, A, L, N, T	CD45+ immume cells	66187 cells from 10XG, 11134 cells from SMART-seq2	scRNA-seq, scTCR repertoire	SMART-seq2, 10X Genomics	Identified 2 distinct macrophage states enriched in HCC tumor tissue and a novel mature DC subtype marked by LAMP3 with potential to migrate to LNs and interact with T/NK cells at tumor site
HNSCC	Puram et al. (2017)	Eighteen treatment naïve patients	L, T	All TME cell types	5902 cells	scRNA-seq, bulk RNA-seq	SMART- seq2	Identified p-EMT program expressed by tumor cells that are spatially localized to the leading edge of primary tumors, predicts metastasis and poor clinical outcomes
HNSCC	Cillo et al. (2020)	Twenty-eight treatment naïve patients (18 HPV-, 8 HPV +), 5 non-cancer, 6 healthy	B, N, T	CD45 + immume cells	131224 cells	scRNA-seq	10X Genomics	Identified differences between CD4 + Tconv lineages in HPV– versus HPV + HNSCC; CD4 + Tconv branches after initial activation into either Tfh or exhausted state; identified heterogeneous myeloid cell populations
Glioma	Müller et al. (2017)	Seven treatment naïve patients (5 GBM, 2 LGG)	T	All TME cell types, CD11b + cells	672 cells from C1, 3132 cells from 10XG	scRNA-seq	10X Genomics, Fluidigm C1	Identified gene signature of the blood-derived tumor associated macrophages (TAMs), which upregulate immunosuppressive cytokines and predicts poor clinical outcomes
Bladder	Oh D.Y. et al. (2020)	Seven patients with diverse treatment background	N, T	CD8, CD4 T cells	30604 cells	scRNA-seq, scTCR repertoire	10X Genomics	Identified cytotoxic CD4 T cells that were clonally expanded in TME and capable of direct killing analogous tumor cells in an MHC-II dependent manner
CRC, NSCLC, renal, endometrial	Wu et al. (2020)	Fourteen treatment naïve patients	B, N, T	CD45 + immune cells or CD3 + T cells	141623 cells	scRNA-seq, scTCR repertoire	10X Genomics	Clonotypic expansion of effector-like T cells in tumor, normal adjacent tissue and peripheral blood; intratumoral tumor-specific T cells are replenished with non-exhausted, newly primed tumor-specific T cells from outside of the TME

#### Cytotoxic CD8^+^ T Cells

**FIGURE 1:**
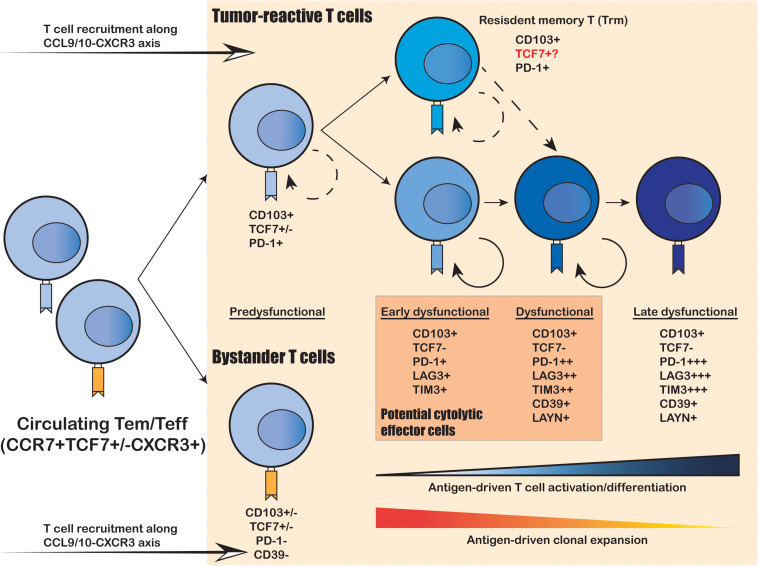
Intratumoral expansion and differentiation of CD8^+^ T cells within the human TME. Upon tumor infiltration, tumor-reactive T cells that recognize tumor-specific antigens undergo antigen-driven T cell activation and differentiation, coupled with antigen-driven clonal expansion. The freshly infiltrated “pre-dysfunctional” tumor-reactive T cells continuously differentiate into various “dysfunctional” states, transitioning from the “early dysfunctional”, “dysfunctional” into the “late dysfunctional” state, characterized by the lack of cytolytic effector capacity and high expression of co-inhibitory markers (e.g., PDCD1, LAG3 and TIM3) as well as T cell exhaustion markers (e.g., CD39 and LAYN). In addition, the tumor-reactive T cells are usually double-positive for tissue-resident T cell marker CD103 and T cell exhaustion marker CD39, whereas the bystander T cells are usually negative for both. Resident-memory-like T cells (Trm) positive for CD103 and PDCD1 also present in the TME and are likely to be tumor-reactive. It remains unclear whether these Trm cells can differentiation into cytolytic tumor-reactive T cells and whether they correspond to the anti-PD-1 responsive TCF7^+^ progenitor-like T cell subset identified in mouse models.

Cytotoxic CD8^+^ T cells are considered as the primary T cell subtype responsible for direct tumor killing. Multiple scRNA-seq studies have demonstrated that intratumoral cytotoxic CD8^+^ T cells are predominantly in the dysfunctional and exhausted state, characterized by their lack of classical effector cytokine secretion and cytolytic functions, as well as their sustained expression of T cell exhaustion markers (Zheng et al., 2017; Azizi et al., 2018; Guo et al., 2018; Zhang et al., 2018; Li H. et al., 2019; Yost et al., 2019; Cillo et al., 2020). Moreover, comprehensive profiling on tens of thousands cytotoxic CD8^+^ T cells has yielded an unprecedented resolution to uncover a continuous spectrum of cell states from the early effector “transitioning” into the fully dysfunctional and exhausted state (Figure 1; Azizi et al., 2018; Li H. et al., 2019; Yost et al., 2019). Therefore, the intratumoral cytotoxic CD8^+^ T cells are not composed of discrete cell subtypes, but a continuum of cell states driven by ongoing activation and differentiation in response to TME stimuli. This level of complexity was under appreciated until the recent technical advances on large-scale single-cell sequencing.

To elucidate the developmental lineages of T cells along the differentiation axis, lineage tracing at the single-cell level is required. Joint analysis of TCR repertoire and transcriptome within a single T cell serves as a powerful tool to connect the phenotype of a given cell with its developmental lineage. Such analyses have revealed that the “dysfunctional” CD8^+^ T cells have undergone the highest level of clonal expansion among the various tumor infiltrating T cell subpopulations (Figure 1; Zheng et al., 2017; Azizi et al., 2018; Guo et al., 2018; Sade-Feldman et al., 2018; Zhang et al., 2018; Li H. et al., 2019; Yost et al., 2019). CD8^+^ T cells can directly recognize tumor cells in an MHC-I-dependent manner to activate the TCR signaling pathway; and then trigger T cell clonal expansion and effector functions to kill the targeted tumor cells via cytolytic activity and secretion of inflammatory cytokines. Therefore, the clonal expansion of intratumoral CD8^+^ T cells reflects their tumor reactivity. On the other hand, several recent studies have unveiled that not all intratumoral CD8^+^ T cells are equally tumor-reactive: the tumor-reactive CD8^+^ T cells are the most clonally expanded subpopulations with a tissue-resident memory phenotype and exhausted phenotype marked by CD103 and CD39, respectively; whereas the bystander CD8^+^ T cells are less clonally expanded and negative for CD103 and CD39 (Figure 1; Duhen et al., 2018; Simoni et al., 2018; Li H. et al., 2019; Scheper et al., 2019). Collectively, the functional state and antigen-specificity are the two key determinants of the anti-tumor capacity of CD8^+^ T cells; and the combination of scRNA-seq and scTCR repertoire analyses enables efficient identification and lineage tracing of tumor-reactive T cells within the human cancer TME.

By applying joint scRNA-seq and scTCR-seq, researchers have generated novel insight into the mechanism underlying PD-1 blockade directly from human specimens. Yost et al. (2019) showed that the majority of CD8^+^ T cells identified from basal cell carcinoma (BCC) and squamous cell carcinoma (SCC) after PD-1 blockade did not share TCRs with the pre-existing intratumoral CD8^+^ T cells, suggesting that the pre-existing intratumoral CD8^+^ T cells had limited capacity of reinvigoration; instead, a fresh population of tumor-reactive CD8^+^ T cells, presumably originated outside of the TME, were recruited to the tumor site in response to PD-1 blockade. Similar observation was made on melanoma, suggesting that the CD8^+^ T cells presented predominately in post-ICB treated tumor samples hardly shared TCRs with the pre-treatment samples (Sade-Feldman et al., 2018). Moreover, despite their lack of reinvigoration capacity, dysfunctional tumor-reactive CD8^+^ T cells can secrete cytokine CXCL13, the ligand for CXCR5 that is commonly expressed on B cells, T follicular helper (T_FH_) cells, and follicular CXCR5-expressing CD8^+^ T cells (Moser et al., 2002; He et al., 2016; Thommen et al., 2018). These findings suggest that in spite of their altered effector functions, these dysfunctional CD8^+^ T cells may play a novel role in recruitment of other immune cells to the TME. Taken together, the recent single-cell studies on human TME has yielded tremendous insight into the functional diversity and developmental lineages of CD8^+^ T cells, advanced our understanding on the mechanism underlying PD-1 blockade, such that the pre-existing tumor-reactive dysfunctional CD8^+^ TILs may have limited reinvigoration capacity following PD-1 blockade, but they may function together with other immune cells (i.e., CD4^+^ T cells, B cells, dendritic cells (DCs), natural killer cells (NKs), etc.) to promote the priming and recruitment of *de novo* tumor-reactive T cells to the tumor. Therefore, although CD8^+^ T cells are the primary tumor-reactive cell type, their infiltration and functionality are largely modulated by other immune cell types, which are also essential for anti-tumor immunity.

#### CD4^+^ Helper T Cells

CD4^+^ T cells are further classified into pro-inflammatory CD4^+^ helper T (T_H_) cells and immunosuppressive CD4^+^ regulatory T (T_REG_) cells. In addition to their well-established role on promoting B cell activation and differentiation, T_H_ cells also promote priming and clonal expansion of antigen-specific CD8^+^ T cells and modulate CD8^+^ T cell-intrinsic effector functionality in the TME and secondary lymphoid organs (Borst et al., 2018). Expansion of an ICOS^+^ T_H_1-like CD4^+^ effector subset was observed in response to CTLA4 blockade; therefore, T_H_ cells, particularly the T_H_1 cells, are also pivotal players in T-cell mediated anti-tumor immunity and can respond to immunotherapy (Wei et al., 2017). Nevertheless, the cellular composition and functional plasticity of intratumoral T_H_ cells are much less well-characterized compared to that of CD8^+^ T cells. Individual scRNA-seq studies have correlated the presence of specific subtypes of T_H_ cells to clinical outcomes in certain cancer types. For example, scRNA-seq on the TME of colorectal tumors identified that intratumoral CXCL13^+^ T_H_1-like cells were enriched in microsatellite-instable (MSI) colorectal tumors, which is associated with significantly higher responsiveness to ICB treatment compared to the microsatellite-stable (MSS) colorectal cancers (Zhang et al., 2018). Moreover, intratumoral CD4^+^ T follicular helper (T_FH_) cells were reported to enrich in HPV^+^ head and neck squamous cell carcinoma (HNSCC) and associate with longer progression-free survival (Cillo et al., 2020). Notably, the formation of tertiary lymphoid structure (TLS) that involves T_FH_ cells and B cells has been recently shown to promote immunotherapy response and survival in multiple cancer types (Thommen et al., 2018; Sautès-Fridman et al., 2019; Cabrita et al., 2020; Helmink et al., 2020; Petitprez et al., 2020). Therefore, CXCL13 expressing CD8^+^ and T_H_1 T cells may work together with T_FH_ cells and B cells on facilitating the priming and recruitment of fresh tumor-reactive T cells to the TME. Additionally, a recent scRNA-seq study on bladder cancer identified two cytotoxic CD4^+^ T_H_ subsets that were clonally expanded and capable of killing analogous tumor cells in an MHC-II-dependent manner. Meanwhile, gene signature of the cytotoxic CD4^+^ T cell subsets predicted clinical response in metastatic bladder cancer patients treated with anti-PD-L1 (Oh D.Y. et al., 2020). Conversely, scRNA-seq analyses on CRC and NSCLC indicated that the most clonally expanded cytotoxic CD4^+^ T cell subpopulation was rather enriched in blood and normal adjacent tissues, suggestive of their migratory feature and potential function outside of the TME (Guo et al., 2018; Zhang et al., 2018). Therefore, the cytotoxicity of CD4^+^ T_H_ cells toward tumor cells is likely cancer type specific, presumably depends on the MHC-II expression level of different tumor cell types.

#### CD4^+^ Regulatory T Cells

In contrast to CD4^+^ helper T cells, which mainly participate in anti-tumor immunity and prevent tumor progression, CD4^+^ regulatory T (T_REG_) cells, characterized by the expression of lineage-specific transcription factor *FOXP3*, are the immunosuppressive T cells correlated with tumor progression and poor clinical outcomes. T_REG_ cells are the key component of immune homeostasis that maintains self-tolerance; and dysfunction in T_REG_ cells often leads to autoimmune diseases (Wing and Sakaguchi, 2010; Togashi et al., 2019). T_REG_ cells exert their immunosuppressive functions through several independent mechanisms including the CTLA-4-mediated suppression of antigen-presenting cells, competition for and consumption of IL-2, secretion of immunosuppressive cytokines such as IL-10 and TGF-β, CD39-dependent conversion of ATP into adenosine, and direct cytotoxic effect on effector cells via granzyme and/or perforin secretion (Togashi et al., 2019). Intratumoral T_REG_ cells are noted as one of the major immune cell types that contribute to the immunosuppressive TME in human cancers. The depletion of intratumoral T_REG_ cells was observed from patients responsive to CTLA4 blockade, whereas the persisted or elevated presence of T_REG_ cells may contribute to ICB resistance (Sharma et al., 2017; Tanaka and Sakaguchi, 2017; Wei et al., 2018; Togashi et al., 2019). scRNA-seq analyses on human cancer TME unveiled phenotypical distinctions of intratumoral T_REG_ cells from circulating and tissue-resident T_REG_ cells, and demonstrated that the intratumoral T_REG_ cells underwent significantly greater clonal expansion compared to the other two T_REG_ populations (Zheng et al., 2017; Guo et al., 2018; Zhang et al., 2018). The clonally expanded TCRs were mostly exclusive to intratumoral T_REG_ themselves, suggesting that they were the tumor-reactive cells locally expanded upon activation by tumor-specific antigens within the TME (Zheng et al., 2017; Guo et al., 2018; Zhang et al., 2018). Furthermore, a considerable level of heterozygosity in terms of functional activation states was observed within the intratumoral T_REG_ population; and only the “activated” T_REG_ expressing high level of both co-inhibitory and co-stimulatory genes correlated with poor prognosis in NSCLC (Guo et al., 2018; Li H. et al., 2019). These findings are in large agreement with another independent functional study demonstrating that the activation and clonal expansion of intratumoral T_REG_ cells within the TME were driven by their tumor antigen reactivity (Ahmadzadeh et al., 2019). Furthermore, through side-by-side scRNA-seq analysis on mouse and human T_REG_ cells, Zemmour et al. (2018) showed that T_REG_ cells could be generally divided into activated/memory and resting/naïve states, and TCR signaling seemed to shape the different facets of activated T_REG_ cells such as their differentiation and effector functions. Therefore, it is conceivable that the functionality of intratumoral T_REG_ cells are shaped by tumor antigens in the TME, contributing to their proliferation and functional plasticity. Whether intratumoral T_REG_ cells were recruited from peripheral blood and/or adjacent non-malignant tissue, or converted from intratumoral T_H_ cells, has not been unequivocally determined. Shared TCR clones were identified from paired analysis of peripheral blood and tumor sample of human breast cancer (Wang et al., 2019), indicating the blood origin of intratumoral T_REG_ cells, whereas bulk RNA transcriptomic analyses on multiple cancer types suggested a closer phenotypic similarity between tumor and tissue-resident T_REG_ cells (Plitas et al., 2016; Magnuson et al., 2018). As compared to blood and tissue-resident T_REG_ cells, intratumoral T_REG_ cells were upregulated for chemokine receptor CCR8 and exhaustion markers such as LAG3, CD39, and LAYN, possibly reflective of their activated and highly immunosuppressive state (De Simone et al., 2016; Plitas et al., 2016; Miragaia et al., 2019; Wang et al., 2019). ScRNA-seq analysis also identified low level of TCR repertoire sharing of intratumoral T_REG_ cells with T_REG_ from both blood and normal mucosa, as well as from intratumoral T_H_ cells, arguing for a potential multi-origin model of tumor T_REG_ cells (Zheng et al., 2017; Guo et al., 2018; Zhang et al., 2018). More extensive single-cell based analyses are necessary to further address the immunosuppressive functions of intratumoral T_REG_ cells derived from different origins to fully understand the mechanism underlying intratumoral T_REG_ cell recruitment, differentiation, and activation. Furthermore, close interaction between intratumoral T_REG_ cells and tumor-associated macrophages (TAMs) was observed from human breast cancer, and this interaction may be important for the development of an immunosuppressive TME (Wang et al., 2019).

Taken together, intratumoral T cells display high level of complexity both in terms of their intrinsic functional states and interactions between other cell subtypes. Single-cell sequencing allowed the identification of “transitional” T cell states, delineation of T cell differentiation trajectory, predication of intercellular interactions, and association of novel T cell subsets with clinical outcomes. These findings significantly enhanced our understanding on T-cell mediated anti-tumor immunity and the mechanism underlying ICB therapy response.

### Tumor Infiltrating Myeloid Cells

In addition to T lymphocytes, tumor infiltrating myeloid cells (TIMs) are also critical mediators of tumor progression and immune evasion. Myeloid cells can modulate T cell functions through their ability to present tumor antigens to T cells and to secrete key cytokines and chemokines for T cell differentiation and recruitment (Fridman et al., 2017; Binnewies et al., 2018). Thus, TIM cell composition appears to modulate lymphocytes infiltration, activation, and their anti-tumor functions. Understanding of the TIM compartment will shed important insight into the regulation of T-cell mediated anti-tumor immunity. However, due to the lack of sufficiently distinctive cell markers and questioned conservation between human and mouse model, human TIM cell composition is much less well-characterized compared to TILs (Zilionis et al., 2019). Single-cell sequencing provides an opportunity to comprehensively profile the TIM compartment independent of pre-defined cell surface markers and assumptions on cross-species conservation, thereby allowing for identification of novel cell lineages and/or cell states, as well as comparison of cell compositions between different species. ScRNA-seq has first been used to identify novel subtypes of DCs, monocytes and progenitor cells from human peripheral blood, scratching the surface of the previously under-appreciated complexity of the myeloid lineage (Villani et al., 2017). Meanwhile, single-cell sequencing is becoming an emerging strategy to validate results from animal models for translation to human immunobiology. For example, a recent scRNA-seq study conducted side-by-side comparison between human and mouse myeloid cell populations from lung cancer TME, and concluded that the population structures of intratumoral DCs, monocytes and neutrophils were largely conserved between mouse and human, whereas that of macrophages exhibited significant difference across species (Zilionis et al., 2019). Knowledge of the human-mouse correspondences and discrepancies of the TIM is tremendously valuable in guiding functional studies on distinct TIM subpopulations using mouse models, and in connecting the abundance of certain TIM subsets with clinical outcomes and therapeutic responses in both human and mouse models.

#### Dendritic Cells

**FIGURE 2:**
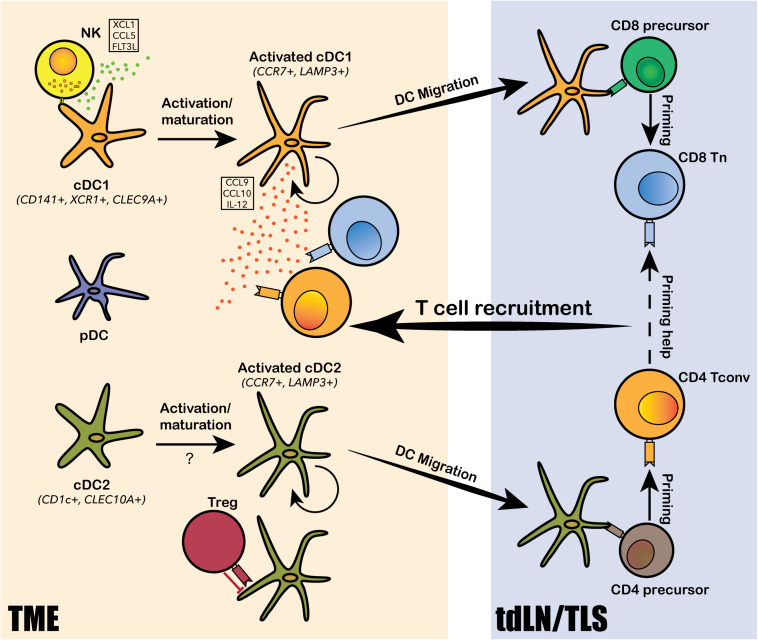
Dendritic cell subtypes identified in the human TME and their functions. Conventional DCs, including the CD141^+^ cDC1 and CD1c^+^ cDC2, and plasmacytoid DCs (pDCs) are identified from the human TME. Both cDC1 and cDC2 undergo intratumoral activation and maturation into “activated” cDC1s and cDC2s, marked by CCR7 and LAMP3. The activated cDCs can present tumor antigens and migrate from the TME to tumor-draining lymph nodes (tdLN) and/or the tertiary lymphoid structure (TLS) to prime T cells for tumor antigen-specific reactivity. cDC1s mainly prime for CD8^+^ T cells and cDC2 mainly prime for CD4^+^ T cells. Tumor-antigen primed T cells are then recruited back to the TME via chemokine axis secreted by the activated cDCs and other immune cells from the TME. DCs also interact extensively with various subtypes of lymphocytes, such as natural killer cells (NKs), regulatory T cells (T_REG_) and CD8^+^ T cells, via a rich array of chemokine and cytokine secretion. NKs can recruit and activate cDC1s by secreting XCL1, CCL5 and FLT3L; TREGs can directly bind to cDC2s and inhibit their migration and priming of CD4^+^ T cells; and cDC1 can secrete IL-12 to modulate CD8^+^ T cell response to PD-1 blockade.

Dendritic cells comprise heterogenous lineages of myeloid cells, including conventional DCs (cDCs), plasmacytoid DCs (pDCs), and monocytes-derived DCs (mo-DCs). The increased abundance of intratumoral cDCs is generally associated with good prognosis, suggesting an anti-tumor role of cDCs (Hashimoto et al., 2011; Veglia and Gabrilovich, 2017; Michea et al., 2018; Moussion and Mellman, 2018). The primary function of cDCs in anti-tumor immunity is to acquire tumor-specific antigens at the tumor site, then migrate to tumor-draining lymph nodes (tdLNs) to prime T cells for *de novo* tumor-specific response. Intratumoral DCs identified from human liver and lung cancers by scRNA-seq consisted of two major subpopulations corresponding to cDC1 and cDC2, a third “activated” cDC subpopulation marked by CCR7 and LAMP3, and a fourth pDC subpopulation derived from a distinct lineage (Figure 2; Lavin et al., 2017; Zhang et al., 2019; Zilionis et al., 2019). cDC1, marked by CD141^+^ in human and CD103^+^ in mouse, excels in cross-presentation and priming of tumor antigens to CD8^+^ T cells; cDC2, marked by CD1c^+^ in human and CD11b^+^ in mouse, mainly primes CD4^+^ T cells. Specific depletion of CD141^+^ cDC1s was observed from early stage lung cancer TME by scRNA-seq and CyToF analyses, suggesting that DC depletion is one of the early hallmark events leading to immune evasion and tumor progression (Lavin et al., 2017). This observation is in line with mouse model studies on CD103^+^ cDC1s, such that the expansion and activation of CD103^+^ DCs dramatically increased CD8^+^ T cell recruitment to the tumor site and transformed response to checkpoint blockade (Roberts et al., 2016; Salmon et al., 2016). More specifically, the CD103^+^CCR7^+^ migratory subset of cDC1 is required for tumor antigen trafficking and priming of CD8^+^ T cells in the tumor draining lymph nodes (tdLN; Roberts et al., 2016; Salmon et al., 2016). The identification of an “activated” cDC subpopulation with predicted migratory capacity in human lung, liver and colorectal cancers further confirmed the conservation between human and mouse in DC biology (Zhang et al., 2019, 2020; Zilionis et al., 2019). The activated CCR7^+^LAMP3^+^ cDCs was comprised of populations derived from both cDC1 and cDC2 cells and interact intensively with T cells and NK cells via a rich array of chemokines and co-stimulatory, co-inhibitory molecules (Zhang et al., 2019, 2020). Two recent studies revealed that NK cells were involved in the recruitment of mouse CD103^+^ cDC1s to the TME through NK cell derived chemoattractants CCL5 and XCL1, and cytokine FLT3LG (Barry et al., 2018; Bottcher et al., 2018). Accordingly, NK cell abundance positively correlated with the presence of cDC1 in human melanoma, increased patient responsiveness to anti-PD-1 therapy, and better clinical outcome (Barry et al., 2018; Bottcher et al., 2018). Notably, the depletion of cDC1 from early stage lung cancer TME was coupled with reduced and impaired NK cells, further supporting the critical role of NK cells in cDC1 recruitment (Lavin et al., 2017). In addition to its role in tumor antigen presentation and CD8^+^ T cell priming, cDC1 was also shown to directly regulate CD8^+^ T cell functions and promote anti-PD-1 therapeutic efficacy through alternative mechanisms. Based on scRNA-seq analysis on mouse models, Garris et al. (2018) showed that intratumoral cDC1s specifically secreted IL-12, which was required for restoring the effector functions of CD8^+^ TILs following PD-1 blockade. Likewise, scRNA-seq analysis on mouse colorectal cancer model MC38 suggested that anti-CD40 agonist specifically activated a subset of cDC1, leading to upregulation of co-stimulatory marker CD80 and CD86, as well as cytokine IL-12 (Zhang et al., 2020). Additionally, cDC1s was reported to secrete chemokines CXCL9 and CXCL10, which interact with intratumoral T-cell expressed receptor CXCR3, to regulate CD8^+^ T cell response to PD-1 blockade (Chow et al., 2019). In addition to studies focusing on cDC1s, a scRNA-seq study on mouse and human tdLN samples identified two subpopulations of migratory cDC2s that were directly responsible for the priming of tumor antigen-specific CD4^+^ T_H_ cells. More importantly, these cDC2 subsets were subjected to suppression by T_REG_ cells in the TME, and the depletion of tumor T_REG_ cells led to enhanced cDC2 migration and generation of functionally matured T_H_1-like cells characterized by ICOS^hi^PD-1^lo^ (Binnewies et al., 2019). Altogether, cDCs play multifaced anti-tumor roles in priming, recruiting and regulating intratumoral T cell differentiation, survival and effector functions.

#### Tumor-Associated Macrophages

**FIGURE 3:**
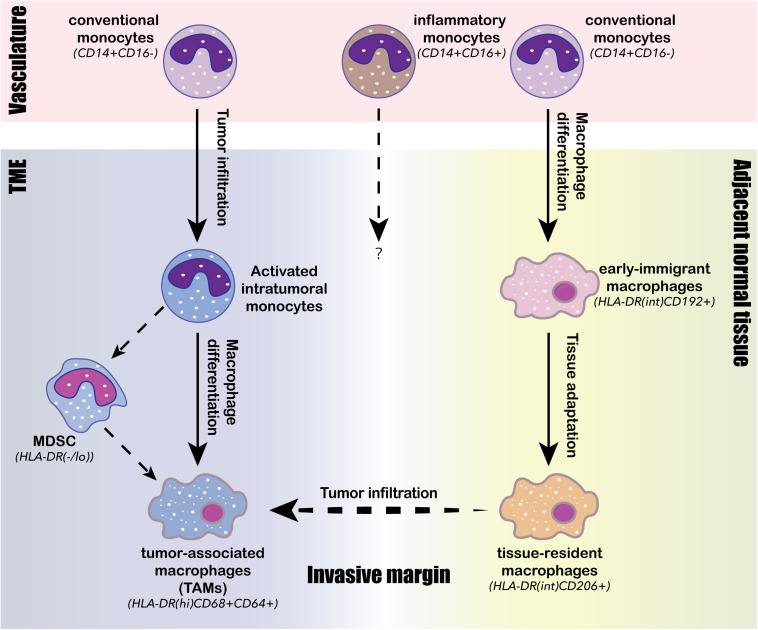
Intratumoral differentiation of the monocyte/macrophage lineage. Three conserved monocyte/macrophage differentiation trajectories are identified from human TME. The first trajectory is the tumor infiltration and activation of conventional monocytes (CD14^+^CD16^–^) from the vasculature into activated intratumoral monocytes; the second trajectory is the ongoing intratumoral macrophage differentiation from the activated intratumoral monocytes into tumor-associated macrophages (TAMs), characterized by high expression of HLA-DR, CD68 and CD64; the third trajectory is the differentiation of circulating conventional monocytes (CD14^+^CD16^–^) into early immigrant macrophages (HLA-DR^int^CD192^+^) and then continue to tissue-resident macrophages (HLA-DR^int^CD206^+^) through stepwise tissue adaptation within the adjacent normal tissue. Tissue-resident macrophages (HLA-DR^int^CD206^+^) can then across the invasive margin to infiltrate tumor and potentially be converted to TAMs (HLA-DR^hi^CD68^+^CD64^+^). The intratumoral monocyte may also differentiate into myeloid-derived suppressor cells (MDSCs), whose presence often negatively associates with clinical outcomes.

In contrast to DCs, the population structure of intratumoral macrophages exhibited considerable human-specific pattern, highlighting the importance on direct analysis of human samples in understanding the pro- and anti-tumor roles of the monocyte/macrophage lineage in human TME (Zilionis et al., 2019). The major monocyte/macrophage lineage-derived cell populations identified in the human TME by scRNA-seq and CyToF analyses consist of classical monocytes (CD14^+^CD16^–^), inflammatory monocytes (CD14^int^CD16^+^), immigrant macrophages (HLA-DR^int^CD192^+^), tissue-resident macrophages (HLA-DR^int^CD206^+^), TAMs (HLA-DR^hi^CD68^+^CD64^+^), myeloid-derived suppressor cells (MDSCs, HLA-DR^–/low^), and monocyte-derived DCs (mo-DCs; Chevrier et al., 2017; Lavin et al., 2017; Azizi et al., 2018; Li H. et al., 2019; Wagner et al., 2019; Zhang et al., 2019; Zilionis et al., 2019; Cillo et al., 2020). Each of these populations can further diversify into a spectrum of activation and differentiation states in response to TME stimuli. Three convergent differentiation trajectories were identified across different cancer types by scRNA-seq analyses: (1) the first trajectory was an ongoing differentiation of monocytes from classical circulating monocytes derived from the tumor vasculature into activated intratumoral monocytes (Azizi et al., 2018; Li H. et al., 2019); (2) the second trajectory was the differentiation of intratumoral monocytes into TAMs (Azizi et al., 2018); and (3) and the third trajectory was the differentiation of classical circulating monocytes into early immigrant macrophages and then into mature tissue-resident macrophages (Figure 3; Chevrier et al., 2017; Wagner et al., 2019). Compared to juxta-tumoral stroma and normal adjacent tissue, tumors were generally enriched for TAMs, intratumoral monocytes and MDSCs, whereas depleted of tissue-resident macrophages, classical circulating monocytes, and inflammatory monocytes (Lavin et al., 2017; Azizi et al., 2018; Li H. et al., 2019; Wagner et al., 2019; Zhang et al., 2019). TAMs comprise a highly heterogenous population derived either from monocytes or tissue-resident macrophages; and they simultaneously express activated anti-tumor M1 markers and pro-tumor M2 markers, suggesting that the classical M1/M2 polarization paradigm may not be applicable to human TAMs (Lavin et al., 2017; Müller et al., 2017; Azizi et al., 2018; Binnewies et al., 2018; Wagner et al., 2019; Zhang et al., 2019). Although the infiltration of TAMs in the TME has been generally associated with tumor progression and poor prognosis, the functional role of TAMs has been controversial and may vary among different cancer types and stages (Keeley et al., 2019). A scRNA-seq study on paired early-stage lung adenocarcinoma and non-cancerous lung tissue identified a tumor-enriched TAM population preferentially expressing high level of *PPAR*γ, *TREM*, *CD81*, *MARCO*, and *APOE*, and was negatively associated with clinical outcome, suggesting a pro-tumor role is this TAM population (Lavin et al., 2017). A separate scRNA-seq study on lung cancer TME identified three macrophage subpopulations strongly associated with poor clinical outcomes, and each of the three subsets expressed a distinct chemokine, presumably interacting with distinct cell types expressing the corresponding receptors within the microenvironment (Zilionis et al., 2019). A third study revealed that TAMs infiltrated in early stage lung cancer TME were highly plastic, co-expressing co-inhibitory and co-stimulatory receptors, suggesting their potential to both stimulate and inhibit T cell functions (Singhal et al., 2019). Although the widespread expression of co-inhibitory ligand PD-L1 in TAMs is thought to involve in the immunosuppression of T cells, two recent studies on early-stage lung cancer suggested otherwise. Singhal et al. (2019) showed that unlike PD-L1 expressing tumor cells, PD-L1^+^ TAMs did not directly suppress T cell functions *in vitro*, but rather protected themselves from T cell mediated cytotoxicity. On the other hand, PD-L1 was also highly expressed in tissue-resident macrophages, and these PD-L1^+^ macrophages accumulated in tight clusters at the tumor invasive margin and negatively correlated with T cell infiltration in tumor (Lavin et al., 2017). Two studies carried out on human breast cancer by scRNA-seq and CyToF analyses identified TAMs with heterogenous expression profiles, phenotypically differed from tissue-resident macrophages in juxta-tumoral regions (Azizi et al., 2018; Wagner et al., 2019). PD-L1 expression was common in these breast cancer infiltrated TAMs, and the presence of PD-L1^+^ TAMs was associated with high grade tumor and poor prognosis, suggesting their role in promoting breast cancer progression (Azizi et al., 2018; Wagner et al., 2019). A scRNA-seq study carried out on human liver cancer identified two distinct states of tumor-enriched macrophages, one of which expressing TAM-like signature genes such as *APOE*, *TREM2*, *GPNMB*, and *SLC40A1*, and the other expressing MDSC-like signatures. Only the TAM-like population was associated with poor prognosis in liver cancer, probably through production of pro-inflammatory cytokines such as TNFα and IL-6 (Zhang et al., 2019). Another recent scRNA-seq study on colorectal cancer also identified two distinct TAM subsets: the *C1QC*^+^ TAMs primarily involved in complement activation and antigen presentation, and the *SPP1*^+^ TAMs primarily involved in tumor angiogenesis and ECM interaction (Zhang et al., 2020). Intriguingly, the two TAM subtypes exhibited differential sensitivity toward CSF1R blockade; and the resistance of the *SPP1*^+^ TAMs against anti-CSF1R treatment may explain the minimal therapeutic benefit observed following anti-CSF1R monotherapy (Zhang et al., 2020). Lastly, a scRNA-seq carried out on human glioma identified two TAM populations with distinct lineages, one corresponding to blood-derived TAMs and the other corresponding to tissue-resident microglial TAMs; and only the blood-derived TAMs was associated with poor clinical outcomes (Müller et al., 2017).

Single-cell sequencing is emerging as an important research tool to directly study the human cancer infiltrated myeloid cell compartment, which heavily relied on mouse models and pre-defined surface markers in the past. These single-cell studies have revealed the expression heterogeneity and functional plasticity of myeloid lineage cells and highlighted their complex crosstalk with lymphocytes. Dendritic cells are crucial for T-cell mediated anti-tumor immunity: they function as the primary tumor antigen-presenting cells for *de novo* priming of tumor-reactive T cells; meanwhile, they secrete a rich array of chemokines, cytokines, and co-stimulatory molecules to modulate T cell functions and their response to ICB therapy (Roberts et al., 2016; Salmon et al., 2016; Garris et al., 2018; Chow et al., 2019). On the other hand, DCs are subjected to intensive regulation by lymphocytes, such as NK cells and TREG cells (Barry et al., 2018; Bottcher et al., 2018; Binnewies et al., 2019). The identification of the “activated” state of cDCs within the human TME by scRNA-seq further validated the capacity of single-cell sequencing in recovering rare transiting cell state (Zhang et al., 2019; Zilionis et al., 2019). Tumor-associated macrophages exhibited the highest cross-species discrepancy among the myeloid lineage, reflected by their divergence from the classical polarization model, and their unique population structure (Müller et al., 2017; Azizi et al., 2018; Zhang et al., 2019; Zilionis et al., 2019). Therefore, understanding of the TAM functions and their clinical relevance to immunotherapy heavily rely on analysis of human samples, attributed by the advances in single-cell sequencing technologies. Nevertheless, current studies on TIMs relying solely on scRNA-seq present several limitations: the lack of spatial resolution limits our understanding on how the spatial organization of immune and stromal cells may impact on immune cell functions and tumor cell invasion; the lack of tracible genetic variations limits the confidence on inferring the developmental lineages of infiltrating myeloid cells; and the lack of direct cell-to-cell interaction information limits our in depth understanding on intercellular crosstalk that modulates the cell localization and functions.

### Tumor Cell Intrinsic Programs and Immunoediting

In addition to immune and stromal cell populations, the tumor cells also play direct and critical roles on regulating immune cell infiltration and their anti-tumor functions (Pitt et al., 2016; Nguyen and Spranger, 2020). Meanwhile, the tumor cells are constantly under selection pressure imposed by the host immune system, and thereby are driven to acquire somatic mutations enabling their escape from immune surveillance, a phenomenon termed as “immunoediting” (Mittal et al., 2014; Galon and Bruni, 2020). Therefore, the interplay between an evolving cancer and a dynamic immune microenvironment is one of the major contributors of tumor evolution. Through scRNA-seq analysis on melanoma patients pre- and post-ICB treatment, Jerby-Arnon et al. (2018) identified an ICB resistance program-upregulation of the CDK4/6 pathway by melanoma tumor cells-that led to T cell exclusion and immune evasion. The authors further demonstrated that ICB therapy shaped the tumor cell expression toward the resistance program, suggestive of immunoediting on tumor cells driven by the restored anti-tumor immunity following ICB treatment. Using similar approach, Puram et al. (2017) identified a partial epithelial-to-mesenchymal transition (p-EMT) program expressed by HNSCC malignant cells localized to the leading edge of primary tumor, driving efficient metastasis. These studies generated novel predictive biomarkers for clinical outcomes and therapeutic responses, highlighting the clinical utility of single-cell sequencing on TME profiling. Tumor-derived chemokines were suggested as another tumor-intrinsic mechanism to modulate the TME: epigenetic silencing of CXCL9/10 was shown to mediate T cell exclusion in mouse ovarian cancer model; and secretion of CXCL1 was reported to recruit myeloid cells and deplete T cells in mouse pancreatic ductal adenocarcinoma (PDAC) model (Peng et al., 2015; Li J. et al., 2018). On the other hand, a series of studies by TRACERx consortium reported a strong correlation between clonal neoantigen burden with T cell infiltration and corresponding intratumoral TCR expansion, suggestive of intensive interplay between the tumor-responsive T cells and their tumor cell targets (McGranahan et al., 2016; Joshi et al., 2019; Rosenthal et al., 2019). Infiltrated T cells exerted strong selection pressure on tumor cells, driving their ongoing immunoediting to achieve immune escape. The major mechanisms include: (1) disruption of the antigen presentation machinery by *HLA* loss of heterozygosity (LOH) or *B2M* loss-of-function mutations; (2) selection for neoantigen depletion by DNA copy number loss, transcriptional repression or epigenetic repression; and (3) mutations in IFN and IL-2 signaling components (Rooney et al., 2015; McGranahan et al., 2016; Joshi et al., 2019; Rosenthal et al., 2019). The aforementioned studies were mainly based on transcriptional profiles of bulk tumor samples, with limited resolution to dissect the cell type specific cancer-immune interplay. Conceivably, the future application of single-cell technologies on this topic will greatly facilitate us to further understand the tumor-intrinsic mechanisms that lead to immune escape and their respective impacts on clinical outcomes.

## Implications of Different Immune Cell Types on Immunotherapy

It has been generally recognized that the abundance of CD8^+^ cytotoxic T cells in TME is associated with favorable clinical outcomes in various cancer types (Fridman et al., 2017); and corresponding biomarker utilizing the CD8^+^/CD3^+^ T cell density ratio has been validated to predict the risk of recurrence for colorectal cancer patients, independent from TNM staging information (Pagès et al., 2018). However, a robust immune-based biomarker, independent from tumor-based PD-L1 level and TMB, has yet been validated in predicting treatment response of immunotherapy. It underscores the importance of identifying immunotherapy-responsive immune cell subsets from the local TME, as well as from the secondary lymphoid organs and peripheral blood. A “progenitor-like” intratumoral CD8^+^ T cell subset that may contribute to the durable response to ICB treatment has been identified in a series of mouse model studies: this Tcf1^+^ CD8^+^ T cell subset is featured by its polyfunctionality to proliferate and differentiate into more cytolytic “terminally exhausted” CD8^+^ T cells in response to PD-1 blockade or TCR stimulation (Im et al., 2016; Utzschneider et al., 2016; Wu et al., 2016; Snell et al., 2018; Chen et al., 2019; Miller et al., 2019; Siddiqui et al., 2019; Yao et al., 2019). TCF1^+^ TILs and CXCR5^+^ TILs have also been observed in human cancer TME; however, whether the human TCF1^+^ or CXCR5^+^ TILs resemble their mouse counterparts remains highly controversial (Brummelman et al., 2018; Sade-Feldman et al., 2018). More interestingly, two mouse model studies utilizing scRNA-seq together with bulk ATAC-seq identified distinct epigenetic signatures between the Tcf1^+^ “progenitor exhausted” and the Tcf1^–^ “terminally exhausted” T cells (Jadhav et al., 2019; Miller et al., 2019). These studies also demonstrated that the epigenetic signatures of different T cell subsets remained stable after PD-1 blockade, underscoring the importance of epigenetic state in regulating T cell fate and function (Jadhav et al., 2019; Miller et al., 2019).

Epigenetic mechanisms, acting in conjunction with transcriptional factors, play a pivotal role in regulation of immune cell differentiation and function. Most importantly, unlike transcriptional profiles that can be reversed upon PD-1 blockade, the epigenetic state of exhausted T cells remained stable, responsible for the failed memory development of the transiently rejuvenated exhausted T cells and their fast re-exhaustion (Youngblood et al., 2011; Ladle et al., 2016; Pauken et al., 2016; Sen et al., 2016; Ghoneim et al., 2017; Mognol et al., 2017). Therefore, the epigenetic imprinting on exhausted or dysfunctional T cells appears to be a major roadblock preventing them from sustainable rejuvenation by ICB treatment. Consistently, distinct epigenetic states corresponded to distinct dysfunctional states of tumor-specific CD8^+^ T cells in tumor mouse model: the “early dysfunctional” TILs obtained a plastic chromatin state that is reprogrammable, whereas the “late dysfunctional” TILs obtained a fixed chromatin state resistant to reprogramming (Schietinger et al., 2016; Philip et al., 2017). This mirrored observations on human TME that tumor-reactive CD8^+^ TILs exhibited various degrees of dysfunction at the transcriptome level, and thereby predicts that the degree of dysfunction might be further sculpted by the epigenetic state which has direct impact on the rejuvenation capacity of exhausted T cells in response to ICB treatment.

In addition to T cells, several recent studies have demonstrated that PD-L1 blockade reinvigorated DC function to enhance T cell priming, and thereby generated potent anticancer T cell immunity (Mayoux et al., 2020; Oh S.A. et al., 2020). A DC gene signature is associated with improved overall survival in patients with renal cancer and NSCLC treated with PD-L1 blockade (Mayoux et al., 2020). Consistent to this view, two recent scRNA-seq studies have observed the expansion of anti-tumor TCR reperitore in the peripheral blood and clonal replacement of intratumoral tumor-specific T cells following PD-1/PD-L1 blockade (Yost et al., 2019; Wu et al., 2020), suggesting the enhanced T cell priming of tumor neoantigens as a major mechanism underlying effective immunotherapy. Altogether, these results have demonstrated the dual-effect of PD-L1/PD-1 blockade on (1) enhancing T cell priming via activation or rejuvantation of DCs and (2) triggering proliferation and differentiation of the intratumoral “progenitor-like” T cells. It predicts that the presence of activated DC subsets and/or proliferative intratumoral T cell subsets would positively associate with the clinical outcome of immunotherapy; and developing biomarkers representing these specific immune cell subsets may yield promising predictive value on immunotherapy response.

## Prospects on Applications of Single-Cell Multi-Omics on TME Studies

Recent applications of scRNA-seq has significantly advanced our understanding on the functional diversity of immune cells in human cancer TME. Moreover, an ongoing activation and differentiation trajectory within the TME was identified from multiple immune cell lineages, such as T cells and macrophages, highlighting the complex crosstalk between the microenvironment and immune cells to regulate their differentiation and function. Single-cell transcriptomics generate a static snapshot of the transcriptional phenotype of individual cells at a single time point. Computational algorithms can be used to further project all sampled cells into a differentiation trajectory based on the assumptions that cells spanning a continuum of transitional states are sufficiently sampled in the dataset, and cells with similar transcriptional profiles are developmentally related (Kester and van Oudenaarden, 2018; Baron and van Oudenaarden, 2019; Tritschler et al., 2019). The most commonly used computational algorithms in TME studies include: (1) Monocle/monocle 2, which projects a minimum spanning tree that connects cells with similar transcriptional profiles and then constructs a “pseudotime” that serves as the backbone of the predicted lineage trajectory (Trapnell et al., 2014; Qiu et al., 2017); (2) and RNA velocity, which predicts the future state of a cell based on the fraction of spliced and unspliced transcripts (La Manno et al., 2018). However, the differentiation trajectories predicted by single-cell transcriptomics data are purely phenotypic, not necessarily reflecting the true genetic relationship between lineages of cells (Kester and van Oudenaarden, 2018; Baron and van Oudenaarden, 2019; Tritschler et al., 2019). Moreover, several key aspects of cellular identity, including but not limited to the epigenetic state, protein profile and spatial location, are missing from the scRNA-seq derived maps. Therefore, moving from scRNA-seq snapshots to multimodal measurements and integration of the genome, epigenome, transcriptome, proteome, and spatial organization datasets will further extend the power of single-cell genomics in immunology studies (Ren et al., 2018; Nathan et al., 2019; Efremova and Teichmann, 2020; Schier, 2020; Zhu et al., 2020).

Growing efforts on developing more sophisticated computational platforms to integrate multimodal single-cell datasets led to more accurate definition of cell identity and state with multiple layers of information. These efforts will ultimately build access into the gene regulatory network that shapes the phenotype and behavior of a pure cell population within complex biological systems (Stuart et al., 2019; Welch et al., 2019). Emerging techniques that allow for simultaneous assessment of multi-omics information from the same cell have also been developed and continue to evolve for greater scalability and higher genomics coverage in the recent years (Table 2; Dixit et al., 2016; Hou et al., 2016; Guo et al., 2017; Peterson et al., 2017; Pott, 2017; Stoeckius et al., 2017; Alemany et al., 2018; Bian et al., 2018; Biddy et al., 2018; Cao et al., 2018; Chen et al., 2018; Clark et al., 2018; Li L. et al., 2018; Satpathy et al., 2018; Spanjaard et al., 2018; Argelaguet et al., 2019; Li G. et al., 2019; Liu et al., 2019; Ludwig et al., 2019; Mimitou et al., 2019; Rodriques et al., 2019; Rooijers et al., 2019; Rubin et al., 2019; Xu et al., 2019; Zhu et al., 2019; Weinreb et al., 2020). For instance, joint analysis of TCR repertoire and chromatin accessibly at the single-cell level was achieved by a novel method named as transcript-indexed ATAC-seq (T-ATAC-seq; Satpathy et al., 2018). By profiling of human peripheral T cells, T-ATAC-seq identified cis- and trans- regulators of naïve and memory CD4^+^ T cell states and substantial epigenomic heterogeneity within the surface-marker-defined T cell populations (Satpathy et al., 2018). This method enables analysis of epigenetic state of clonal T cells, which will be particularly useful for lineage tracing of clonal T cells undergoing differentiation to understand the epigenetic regulation of T cell differentiation and memory formation in response to different environmental stimulations.

**TABLE 2:** Recent advances in single-cell multimodal sequencing technologies.

Method	Multi-omics	Literature	Application	Throughput
T-ATAC-seq	ATAC + RNA	Satpathy et al. (2018)	Immune profiling	Low
scCAT-seq	ATAC + RNA	Liu et al. (2019)	Embryonic development	Low
sci-CAR	ATAC + RNA	Cao et al. (2018)	NA	High
Paired-seq	ATAC + RNA	Zhu et al. (2019)	Brain development	High
scNMT-seq	Methylation + ATAC + RNA	Clark et al. (2018); Argelaguet et al. (2019)	Embryonic development	Low
scTrio-seq	Methylation + RNA + DNA copy number	Hou et al. (2016); Bian et al. (2018)	Tumor heterogeneity	Low
scCOOL-seq	Methylation + ATAC + DNA copy number	Guo et al. (2017); Li L. et al. (2018)	Embryonic development	Low
scNOMe-seq	Methylation + ATAC	Pott (2017)	Technology development	Low
Methyl-Hi C	Methylation + Hi C	Li G. et al. (2019)	NA	Low
CITE-seq	RNA + epitope	Stoeckius et al. (2017)	Immune profiling	High
Pi-ATAC	RNA + epitope	Chen et al. (2018)	Immune profiling	Low
REAP-seq	RNA + epitope	Peterson et al. (2017)	Immune profiling	High
ECCITE-seq	RNA + epitope + CRISPR	Mimitou et al. (2019)	Immune profiling	High
Slide-seq	Spatial + RNA	Rodriques et al. (2019)	Brain development	High
scDam&T-seq	Protein-DNA contacts + RNA	Rooijers et al. (2019)	NA	Low
Perturb-seq	RNA + CRISPR	Dixit et al. (2016)	Immune cell differentiation	High
Perturb-ATAC	ATAC + CRISPR	Rubin et al. (2019)	Keratinocyte differentiation	Low
ScarTrace	RNA + CRISPR	Alemany et al. (2018)	Lineage tracing	Medium
LINNAEUS	RNA + CRISPR	Spanjaard et al. (2018)	Lineage tracing	High
LARRY	RNA + exogenous barcode	Weinreb et al. (2020)	Lineage tracing	High
CellTagging	RNA + exogenous barcode	Biddy et al. (2018)	Lineage tracing	High
EMBLEM	ATAC + mtDNA	Xu et al. (2019)	Lineage tracing	Low
	ATAC + mtDNA	Ludwig et al. (2019)	Lineage tracing	Low

### Lineage Tracing

The joint single-cell analysis of TCR repertoire and transcriptome has yielded tremendous insight into T cell differentiation process by connecting the genetic lineage of a given T cell reflected by its highly polymorphic TCR sequence to its functional phenotype defined by the transcriptome. A landmark study applied this approach to delineate the complete T cell differentiation roadmap of human, from early progenitors residing in the hematopoietic fetal liver and thymus into fully matured T cell types, via sampling of developing human thymus ranged from the early embryonic stage to the adulthood (Park et al., 2020). In the context of human TME, similar analyses have unveiled that a shared TCR clonotype can span a wide range of T cell differentiation states, supporting that these cell states are in fact developmentally linked (Guo et al., 2018; Zhang et al., 2018; Li H. et al., 2019). Conversely, certain TCR clonotypes are almost restricted to a specific cell subset, suggestive of its distinct developmental lineage (Guo et al., 2018; Zhang et al., 2018; Li H. et al., 2019; Yost et al., 2019). Despite of these important observations, our direct knowledge on the developmental lineages of immune cells in human TME remains very limited. For instance, the origin of intratumoral T_REG_ cells remains elusive since vast majority of their TCR clonotypes are exclusive to themselves, with only a small fraction of TCR sequences shared with T_REG_ cells from blood or adjacent normal tissue, or with intratumoral T_H_ cells (Guo et al., 2018; Zhang et al., 2018). Therefore, it remains largely unknown whether intratumoral T_REG_ cells are primarily derived from blood T_REG_, tissue-resident T_REG_ or intratumoral T_H_ (iT_REG_), and whether intratumoral T_REG_ cells derived from different origins exert distinct functions. Another important but unanswered question is the cell origin of the expanded T cell population in response to PD-1 blockade. Two groups have independently observed the “clonotype replacement” phenomenon following responsive anti-PD-1 treatment in skin cancer patients: clonotypes expanded after PD-1 blockade were different from the pre-existing clonotypes identified from the pre-treatment TME, suggesting limited reinvigoration capacity of the majority pre-existing T cells in the TME (Sade-Feldman et al., 2018; Yost et al., 2019). However, it remains undetermined whether these expanded T cell clones are originated from newly primed T cells outside of the TME, or from clonal expansion and differentiation of pre-existing “progenitor-like” T cells that are previously undetectable due to their rarity.

Prospective lineage tracing enabled by simultaneous profiling of single-cell transcriptome and the unique DNA barcodes introduced by genetic manipulation allows for tracking the clonal dynamics of different cell lineages by coupling the transcriptionally defined cell differentiation states with the perspective clonal identifiers that record the past history of cells (Biddy et al., 2018; Spanjaard et al., 2018; Weinreb et al., 2020). Weinreb et al. (2020) applied this approach to study the fate determination in hematopoiesis and precisely identified a continuous spectrum of cell states with primed fate potential, demonstrating the superior performance of this multimodal system compared to the previously used single-modal scRNA-seq only or clonal barcoding only systems. Moreover, this study has drawn two important conclusions that are instrumental to future lineage tracing studies: (1) matured cells derived from distinct lineages are differentially imprinted and thereby phenotypically distinct; (2) transcriptome alone is not sufficient to define the fate potential of progenitor cells, suggesting other heritable properties are missing from the current system, presumably including the epigenetic states, protein abundances, cell organizations, and the microenvironment (Weinreb et al., 2020). Despite of its robustness, prospective lineage tracing can be only applied to *in vitro* and animal model systems due to the requirement of genetic manipulation. Instead, retrospective lineage tracing, taking advantage of the naturally occurring somatic mutations during development, has been widely used for analysis of human samples (Woodworth et al., 2017). A variety of inheritable genetic alternations have been used as the natural “DNA barcodes”, such as TCR/BCR sequences (Han et al., 2014; Stubbington et al., 2016), copy number variations (CNVs; McConnell et al., 2013; Cai et al., 2014), single nucleotide variations (SNVs; Lodato et al., 2015), retrotransposon elements (i.e., LINE-1; Evrony et al., 2012), microsatellite repeats (Evrony et al., 2012, 2015) and mitochondrial DNA (mtDNA; Ludwig et al., 2019; Xu et al., 2019). Lineage tracing based on mtDNA mutations has been recently applied to study the lineage of macrophages in human liver cancer TME, and generated consistent results with independent RNA velocity-based prediction (Zhang et al., 2019). Conceivably, with the emergence and further adoption of single-cell multimodal sequencing technologies, lineage tracing analysis on immune cell populations from mouse and human TME will generate important insight into the regulatory mechanisms underlying immune cell differentiation, exhaustion and memory formation in response to local TME stimuli and various types of immunotherapy.

### Current Limitations and Challenges of Single-Cell Multiomic Technologies

Despite of its great potential, the current single-cell technologies suffer from their limited scalability, sparse coverage, allelic dropouts, PCR errors, and most importantly, lack of spatial information (Nam et al., 2020). For instance, the current scRNA-seq technologies typically generate hundreds to thousands of cells per biological sample, and thousands to hundred thousands of cells per study. Compared to FACS and CyToF analyses, which typically generate data from millions of cells, accurate quantification of cell subsets, especially for rare subsets, remains a challenge for current single-cell technologies. Fortunately, the recent development of combinatorial indexing-based single-cell technology holds promise to further extent the scalability of current single-cell technologies by 1 to 2 orders of magnitude without increase in cost (Cao et al., 2017, 2018; Rosenberg et al., 2018; Zhu et al., 2019), and thereby approaching the throughput of conventional FACS and CyToF methods. Furthermore, since current single-cell sequencing technologies mainly rely on front-end molecular amplification of the picogram scale genomic content from individual cells, they inevitably suffer from PCR errors and allelic dropouts. Meanwhile, due to the limited genomic amplification efficiency and high sequencing cost, the genomic coverage of each individual cell is often very sparse, resulting technological challenges in data processing and interpretation. Therefore, specialized bioinformatic algorithms are necessary to compensate the errors and missing information associated with the typical single-cell sequencing datasets, limiting the general accessibility of these technologies to the greater research community. Last but most importantly, the current single-cell technologies often require front-end tissue dissociation, which inevitably destroys the spatial architecture of the biological specimens, eliminating a critical layer of information that contributes to the biological identity of a cell. To address this limitation, spatial sequencing techniques, such as Slide-Seq (Rodriques et al., 2019), have been developed; and the data integration of spatial transcriptomics is hopeful to help dissect important cell interactions at single-cell resolution in the near future (Adey, 2019; Welch et al., 2019; Nam et al., 2020).

## Conclusion Remarks

Recent technological advancements in single-cell genomics have greatly impacted the way immunologist to conduct their research, particularly in the immune-oncology field. Due to the highly heterogeneous nature of the immune system, single-cell based analysis such as FACS has always been the gold standard for immunologists to study the phenotypes of immune cells. However, conventional FACS analysis is limited by the number of markers that can be simultaneously analyzed and is largely restricted to analysis of surface markers. Although the development of CyToF largely increased the number of markers to be assessed in parallel, it remains constrained by the availability of known surface markers, which limits the discovery of previously unknown cell states. The emergence of scRNA-seq has revolutionized the way immunologists to “phenotype” their cellular population of interest: instead of assigning a cell into a particular cell type positive or negative for a set of pre-defined markers, scRNA-seq places a cell into a phenotypic continuum reflecting various aspects of its cellular identity including activation, differentiation, metabolic states and more. This leads to the identification of immune cells with much more functional diversity than previously appreciated. Moreover, scRNA-seq unveiled that immune cells differentiate along a continuous trajectory attributed by intrinsic regulatory network and extrinsic environmental stimuli, instead of differentiating step-wisely into discrete cell types or intermediates defined by selected markers. Additionally, the development of single-cell multimodal sequencing enables simultaneous profiling of genome, epigenome, transcriptome, proteome and spatial localization from the same cell (Guo et al., 2017; Peterson et al., 2017; Stoeckius et al., 2017; Chen et al., 2018; Clark et al., 2018; Li L. et al., 2018; Argelaguet et al., 2019; Mimitou et al., 2019). Combining genome, epigenome and transcriptome from the same cell allows for retrospective lineage tracing directly from human samples, which is critical for the understanding of immune cell differentiation, activation and exhaustion in response to TME stimuli. Combining epigenome, transcriptome and proteome allows for more accurately defining the cell identity from multiple layers to fully elucidate the functional plasticity of a cell, as well as its past history and future potential. Addition of the spatial localization to the molecular profiles further complements the definition of cell identity, which is also regulated by spatial position for its normal function. Single-cell multi-omics, in a spatially resolved context, is important for understanding the interactions between cells of the immune system and crosstalk between tumor and other cell types in the TME. For instance, the TCR spatial heterogeneity, directed by the physical interaction between TCR and its targeting neoantigen, reflects genomic intratumoral heterogeneity (Joshi et al., 2019); and the spatial distribution of intratumoral PD-L1^+^ macrophages impacts T cell infiltration (Lavin et al., 2017). Lastly, the integration of large-scale datasets across platforms, omics and species, and combination with relevant functional and clinical information, will ultimately transform our understanding of human anti-tumor immunity. This will hopefully allow for improved patient stratifications, biomarker discovery and druggable targets identification, leading to the achievement of precision immunotherapy with better efficacy and less toxicity (Giladi and Amit, 2018).

## Author Contributions

XC conceived the project. TG and XC wrote the manuscript with input from WL. All authors read and approved the final manuscript.

## Conflict of Interest

The authors declare that the research was conducted in the absence of any commercial or financial relationships that could be construed as a potential conflict of interest.
